# Novel fimbrilin PGN_1808 in *Porphyromonas gingivalis*

**DOI:** 10.1371/journal.pone.0173541

**Published:** 2017-03-15

**Authors:** Keiji Nagano, Yoshiaki Hasegawa, Yasuo Yoshida, Fuminobu Yoshimura

**Affiliations:** Department of Microbiology, School of Dentistry, Aichi Gakuin University 1–100 Kusumoto-cho, Chikusa-ku, Nagoya, Aichi, Japan; University of the Pacific, UNITED STATES

## Abstract

*Porphyromonas gingivalis*, a periodontopathic gram-negative anaerobic bacterium, generally expresses two types of fimbriae, FimA and Mfa1. However, a novel potential fimbrilin, PGN_1808, in *P*. *gingivalis* strain ATCC 33277 was recently identified by an *in silico* structural homology search. In this study, we experimentally examined whether the protein formed a fimbrial structure. Anion-exchange chromatography showed that the elution peak of the protein was not identical to those of the major fimbrilins of FimA and Mfa1, indicating that PGN_1808 is not a component of these fimbriae. Electrophoretic analyses showed that PGN_1808 formed a polymer, although it was detergent and heat labile compared to FimA and Mfa1. Transmission electron microscopy showed filamentous structures (2‒3 nm × 200‒400 nm) on the cell surfaces of a PGN_1808-overexpressing *P*. *gingivalis* mutant (deficient in both FimA and Mfa1 fimbriae) and in the PGN_1808 fraction. PGN_1808 was detected in 81 of 84 wild-type strains of *P*. *gingivalis* by western blotting, suggesting that the protein is generally present in *P*. *gingivalis*.

## Introduction

*Porphyromonas gingivalis*, a gram-negative anaerobic coccobacillus, is a member of the “red complex” of bacteria, which are pathogens primarily responsible for human periodontal diseases [1]. The pathogen colonizes the gingival crevice by forming a biofilm with multiple species of bacteria and associating with gingival tissues, leading to a niche of dysbiotic microbiota [2]. Colonization by this bacterium is regulated largely by fimbriae, which are filamentous structures expressed on bacterial cell surfaces [3].

*P*. *gingivalis* generally expresses two distinct types of fimbriae called FimA and Mfa1 [4]. FimA fimbriae are primarily composed of polymers of the FimA protein, encoded by the *fimA* gene [5, 6]. FimB (encoded by *fimB*) localizes in the outer membrane, helping to anchor and regulate the length of FimA fimbriae [7]. FimC, FimD, and FimE (encoded by *fimC*, *fimD*, and *fimE*, respectively) are incorporated into the fimbriae as minor accessory components and may function as adhesins [8]. The *fimA*‒*E* genes are arranged in tandem in a gene cluster on the bacterial chromosome. Similarly, the Mfa1 protein (encoded by *mfa1*) polymerizes to form fimbrial filament, Mfa2 (encoded by *mfa2*) functions as a fimbrial anchor and length regulator, and Mfa3, Mfa4, and Mfa5 (encoded by *mfa3*, *mfa4*, and *mfa5*) are integrated into the fimbriae as minor accessories [9, 10]. The *mfa1*‒*5* genes also form a gene cluster. At least five genotypes of *fimA* have been identified [11], and the fimbriae of these genotypes show differential antigenicities [12]. In addition, a variant of Mfa1 fimbriae, previously called 53-kDa fimbria [13], has been observed [14]. Thus, there are FimA and Mfa1 variants. However, another type of fimbriae has not been identified in *P*. *gingivalis*.

Recently, Xu *et al*. reported the crystal structures of component proteins of FimA and Mfa1 fimbriae [15]. They also reported a potential fimbrial protein, PGN_1808, in *P*. *gingivalis* ATCC 33277, based on an *in silico* structural homology search. In this study, we experimentally examined whether this protein was expressed as a fimbria in *P*. *gingivalis*.

## Methods

### Bacterial strains and culture conditions

We used *P*. *gingivalis* strain TDC60 [16] and several mutants derived from *P*. *gingivalis* strain ATCC 33277 [17] (as described below). We also used additional *P*. *gingivalis* wild-type strains (S1 Table in Supporting Information) [12]. *P*. *gingivalis* was maintained on Brucella HK Agar (Kyokuto Pharmaceutical Industrial Co., Ltd, Tokyo, Japan) supplemented with 5% laked rabbit blood at 37°C under anaerobic conditions. When *P*. *gingivalis* cells were used for experiments, they were cultivated in GAM broth, Modified (Nissui Pharmaceutical Co., Ltd, Tokyo, Japan) and collected at early stationary phase. When needed, 20 μg/mL erythromycin, 10 μg/mL chloramphenicol, and 1 μg/mL tetracycline were added to the medium. *Escherichia coli* strains DH10B, BL21 (DE3), and S17-1 were used for cloning, expressing the recombinant protein, and transferring the shuttle vector to *P*. *gingivalis* through conjugation, respectively [18]. *E*. *coli* was cultivated in Luria-Bertani medium at 37°C. When needed, 100 μg/mL ampicillin, 50 μg/mL kanamycin and 10 μg/mL tetracycline were added to the medium.

### Overexpression of the PGN_1808 protein in *P*. *gingivalis*

Hereafter, we use terms *pgn_1808* and PGN_1808 as the gene and protein names, respectively. We constructed a PGN_1808-overexpressing *P*. *gingivalis* by introducing *pgn_1808* through a plasmid vector into a *P*. *gingivalis* mutant derived from strain ATCC 33277 which is deficient in both Mfa1 and FimA fimbriae because of deletion of the *mfa1* gene and entire *fim* gene cluster (*P*. *gingivalis fimA*^-^ *mfa1*^-^)[18]. We used the shuttle vector plasmid pT-COW with the strong *P*. *gingivalis ragA* promoter (pT-COW_*PragA*_) [19]. The *pgn_1808* coding sequence was amplified by polymerase chain reaction (PCR) from the chromosomal DNA of *P*. *gingivalis* ATCC 33277 using primers 5′-TCAAATTTTATCTAGAATGCTTACGAAACTAAAAACACTG-3′ and 5′-TTGTCTCTCAGCGGCCGCCTACAGATCCACGTCCTGCTC-3′, with restriction enzyme recognition sites, *Xba*I and *Not*I, respectively, incorporated (underlined). The PCR product was digested by the restriction enzymes, ligated to pT-COW_*PragA*_, which was digested by the same enzymes immediately downstream of the *ragA* promoter, and introduced into *E*. *coli* DH10B followed by *E*. *coli* S17-1. The absence of unintended mutations in the *pgn_1808* gene was confirmed by DNA sequencing. Then, the plasmid bearing *pgn_1808* was transferred from *E*. *coli* S17-1 to *P*. *gingivalis fimA*^-^ *mfa1*^-^ through conjugation.

### Preparation of anti-PGN_1808 antiserum

We cloned *pgn_1808* with the 5′-terminal 66 nucleotides encoding a predicted signal peptide removed. We could not obtain the entire PGN_1808 from *E*. *coli*, possibly because of harmful effects. The truncated *pgn_1808* was amplified by PCR from the chromosomal DNA of *P*. *gingivalis* ATCC 33277 using the primers 5′-GGATTTTCGGATCCGAATCATCCGGTATTGACGAATG-3′ and 5′-GTCTCTCAGACTCGAGCTACAGATCCACGTCCTGCTC-3′, with restriction enzyme recognition sites, *BamH*I and *Xho*I, respectively, incorporated (underlined). The DNA fragment was ligated to the pET28 (b) plasmid (Novagen, Darmstadt, Germany), which was digested by the same enzymes to append a hexa-histidine tag to the N-terminus of truncated PGN_1808. The resultant plasmid was then introduced into *E*. *coli* DH10B followed by *E*. *coli* BL21 (DE3). The absence of unintended mutations in the target gene was confirmed by DNA sequencing. After isopropyl-β-D-thiogalactopyranoside induction, the bacterial cells were collected and lysed in BugBuster HT Protein Extraction Reagent (EMD Millipore Corp., Billerica, MA, USA). The soluble fraction was isolated from the lysate by centrifugation at 20,000 × g for 15 min. The histidine-tagged, truncated PGN_1808 was purified using a cobalt-affinity column, emulsified with complete Freund’s adjuvant, and injected into a rabbit to elicit an anti-PGN_1808 antiserum.

### Fractionation of bacterial lysates and fractionation of PGN_1808, FimA, and Mfa1 fimbriae

*P*. *gingivalis* TDC60, normally expressing both FimA and Mfa1 fimbriae, was used for cellular fractionation. The PGN_1808-overexpressing *P*. *gingivalis* (described above) was used for fractionation of PGN_1808. The *mfa1* and *fimA* gene-deletion mutants derived from *P*. *gingivalis* ATCC 33277 were used for the purifications of FimA and Mfa1 fimbriae, respectively [14].

Fractionation were performed largely according to a previously described method [5]. Briefly, *P*. *gingivalis* cells were collected from 1 L of culture, suspended in 40 ml of 20 mM Tris/HCl buffer at pH 8.0, supplemented with protease inhibitors [1 mM phenylmethylsulfonyl fluoride and 1 mM *N*α-*p*-tosyl-_L_-lysine chloromethyl ketone], and disrupted using a French press at 7.3 MPa. Unbroken cells and large debris were removed by centrifugation. The supernatant was subjected to precipitation with 50% ammonium sulfate saturation. The precipitate was dialyzed with the Tris buffer, and then applied to DEAE Sepharose Fast Flow chromatography (GE Healthcare Bio-Sciences AB, Uppsala, Sweden) with 50 ml of bed volume. After washing thoroughly with the buffer, sample was fractionated by a linear gradient elution with 400 ml of NaCl (0 to 0.5 M) in the buffer.

### Transmission electron microscopy

Intact bacterial cells or the PGN_1808 fraction as described above was placed on a grid with an elastic carbon supporting film (Okenshoji Co., Ltd., Tokyo, Japan), negatively stained with 10 mM ammonium molybdate, pH 7.0, and observed by transmission electron microscopy (TEM, JEM-1210, JEOL, Tokyo, Japan).

### Slide agglutination assay

The slide agglutination assay was performed following a standard protocol. *P*. *gingivalis* strains were washed with PBS, pH 7.4, and the optical density at 600 nm was adjusted to 0.5, 1.0 and 2.0. The bacterial suspensions were mixed with the antisera to PGN_1808 or whole cells of *P*. *gingivalis* cells [12] on a glass slide.

### Sodium dodecyl sulfate-polyacrylamide gel electrophoresis (SDS-PAGE), protein staining, and western blot analyses

Samples were mixed with a loading buffer consisting of 50 mM Tris/HCl, pH 6.8, 1% (w/v) SDS, 0.5 M 2-mercaptoethanol, 10% (w/v) glycerol, and 0.01% (w/v) bromophenol blue (all at final concentrations), and denatured by heating at the indicated temperatures for 10 min. Then, the samples were loaded onto an SDS-PAGE gel consisting of 11% or 5‒20% gradient polyacrylamide. After electrophoresis, protein bands were visualized by staining with Coomassie Brilliant Blue (CBB) R-250. For the western blot analysis, proteins separated in the SDS-PAGE gel were transferred to a nitrocellulose membrane. The membrane was blocked with 5% skim milk in 20 mM Tris/HCl, pH 7.5, supplemented with 300 mM NaCl and 0.05% (w/v) Tween 20, and incubated with the primary anti-FimA [18], anti-Mfa1 [9], and anti-PGN_1808 antisera (as described above); all were diluted 1:5,000. These antisera did not show any cross-reactivity (data not shown). After washing, the membrane was incubated with a peroxidase-conjugated secondary antibody at dilution of 1:5,000. Bands were detected using Western BLoT Chemiluminescence HRP Substrate (Takara Bio Inc.).

### Blue native-PAGE

Fractionated PGN_1808 was applied to blue native-PAGE [20]. Briefly, PGN_1808 in Bis-Tris/HCl, pH 7.0 containing 0.5% CBB G-250, 50 mM 6-aminocaproic acid, 0.1 mM PMSF, and 10% glycerol was loaded onto a 4‒10% acrylamide gel supplemented with 1 M 6-aminocaproic acid. Electrophoresis was performed with cathode (50 mM Tricine/NaOH, pH 7.0, 15 mM Bis-Tris, pH 7.0, and 0.02% CBB G-250) and anode (50 mM Bis-Tris, pH 7.0) buffers at 4°C. When the leading edge of the sample reached a half-way point of the gel, the cathode buffer was exchanged for one without CBB G-250. After electrophoresis, the gel was subjected to western blotting with anti-PGN_1808 antiserum.

### Gel filtration chromatography

The molecular size of the fractionated PGN_1808 was examined by using HPLC with a gel filtration chromatography column TSKgel G3000SW_XL_ (7.8 × 300 mm; Tosoh Corp., Tokyo, Japan) at a flow rate of 1.0 ml/min in PBS, pH 7.4, supplemented with 0.02% NaN_3_. The protein elution was monitored by optical absorbance at 280 nm.

### Mass spectrometry and protein sequencing analyses

Mass spectrometry analysis was performed as described previously [14]. N-terminal protein sequencing was performed using a Procise 492HT sequencer (PerkinElmer, Inc., Waltham, MA, USA) [21] at the Open Facility, Hokkaido University Sousei Hall.

### Analysis of transcription unit

Transcription unit comprising *pgn_1808* was examined by reverse transcription (RT)-PCR. Total RNA was isolated using ZR Fungal/Bacterial RNA MiniPrep (Zymo Research Corp., Irvine, CA, USA) and treated with DNase I (Zymo Research Corp.) to remove residual DNA strands. No genomic DNA contaminants were detected in the total RNA samples (data not shown). The pure RNA was used to generate cDNA with a PrimeScript RT-PCR Kit and random 6 mers (Takara Bio Inc., Otsu, Japan). Transcription unit was examined by a standard PCR for intergenic regions using the cDNA as a template and primers listed in S1 Table.

### Bioinformatics

The LipoP 1.0 online program (http://www.cbs.dtu.dk/services/LipoP/) was used for predictions of lipoprotein signal peptides [22]. *In silico* structure homology-modeling of PGN_1808 was performed using the SWISS-MODEL online program (https://swissmodel.expasy.org/) [23].

## Results and discussion

### Fractionation of cell lysates

Previously, we detected PGN_1808 concomitantly with FimA and Mfa1 fimbriae when the fimbriae were fractionated by ammonium sulfate precipitation and anion-exchange chromatography [[24] and unpublished data]. Therefore, we first examined whether the protein was a component of the FimA and Mfa1 fimbriae. We used *P*. *gingivalis* strain TDC60 for the fractionation assay because TDC60 normally expresses both FimA and Mfa1 fimbriae [12], whereas the type strain *P*. *gingivalis* ATCC 33277 expresses aberrant FimA fimbriae due to a nonsense mutation in *fimB* [7].

**Fig 1:**
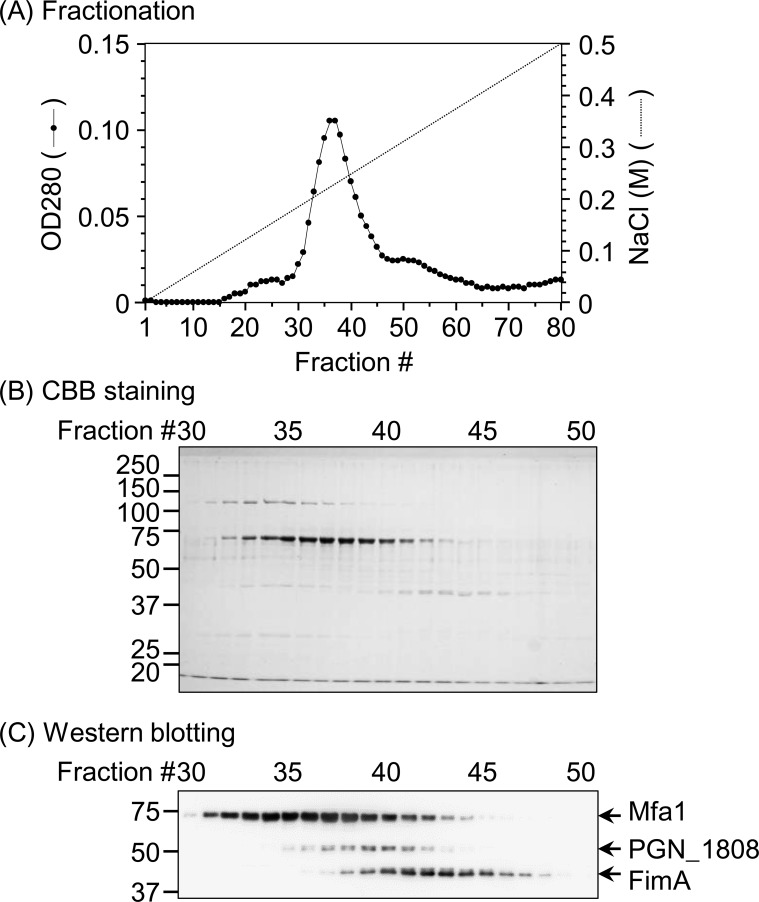
Fractionation of bacterial cell lysates. *P*. *gingivalis* TDC60 lysates were fractionated by anion-exchange chromatography after ammonium sulfate precipitation (A). Fractions 30 to 50 (10 μl each) were subjected to SDS-PAGE, followed by CBB staining (B) and western blotting using anti-FimA, anti-Mfa1, and anti-PGN_1808 antisera (C). We demonstrated reproducibility by repeating the fractionation experiment three times (data not shown). Numbers in left indicate molecular weights (kDa).

The elution diagram showed a major peak at fractions 36 and 37 (Fig 1A), which was almost consistent with the elution behavior of Mfa1 (Fig 1B and 1C), suggesting that Mfa1 was a major protein in strain TDC60. FimA was the most strongly detected at fraction 42 (Fig 1C). Fig 1B and 1C show that the 70-kDa bands corresponding to Mfa1 (fractions 32 to 44) were much more intense than the 40-kDa bands corresponding to FimA (fractions 39 to 48), indicating that Mfa1 was a major fimbria in this strain. The most intensive band of PGN_1808 was shown at fraction 39 (Fig 1C). Thus, the elution peak of PGN_1808 was different from that of the FimA and Mfa1 proteins, indicating that PGN_1808 was not a component of either FimA or Mfa1 fimbriae.

### Electrophoretic and N-terminal sequencing analyses

**Fig 2:**
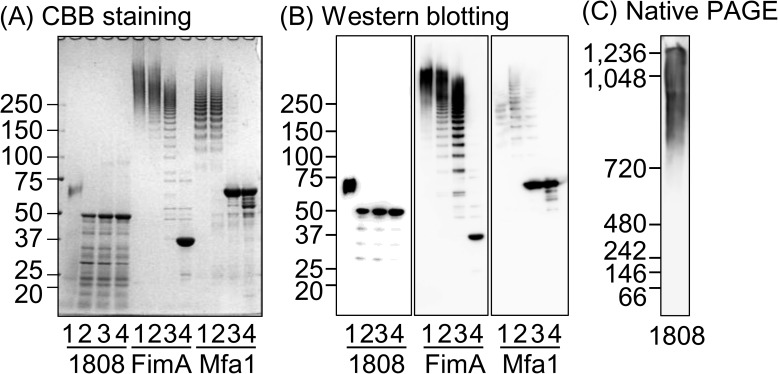
Electrophoretic analyses of PGN_1808 polymerization. Fractionated products of PGN_1808 (1808), FimA and Mfa1 in SDS-containing buffer were heated at 37 (1), 60 (2), 80 (3) and 100 (4)°C for 10 min, and then applied to SDS-PAGE. The SDS-PAGE gel was stained with CBB (A), and western blotting was performed with anti-PGN_1808, anti-FimA, and anti-Mfa1 antisera (B). Fractionated PGN_1808 was applied to blue native-PAGE followed by western blotting (C). Samples containing 5, 2 and 20 μg of proteins were applied to the experiments in panels A, B, and C, respectively. Numbers in left indicate molecular weights (kDa). Note that anti-Mfa1 antiserum weakly reacts to ladder-like band at high molecular weight (Mfa1 polymer) when compared to its monomer, resulting in the differential intensity of bands between CBB and western blot analyses.

Denaturation of FimA and Mfa1 fimbriae by heating at low temperatures (37, 60, and/or 80°C) resulted in a smear and/or ladder-like pattern, due to partial dissociation of the FimA and Mfa1 polymers, respectively, in the SDS-PAGE, whereas denaturation by heating at 100°C resulted in a single band at the sizes corresponding to their monomers (FimA at 40 kDa and Mfa1 at 70 kDa), although degraded products of these proteins were also detected (Fig 2A and 2B), as previously reported [5, 18, 25]. In the PGN_1808 fraction, only a major band at 50 kDa, corresponding to its monomer, was detected following denaturation by heating at 60°C or higher, although treatment at 37°C resulted in a smear at around 70 kDa, presumably due to insufficient denaturation. Visible bands were noticed at a lower molecular weight than the monomer and were identified as degradation products of PGN_1808 by mass spectrometry and N-terminal sequencing analyses. In blue native-PAGE followed by western blotting, the smear signal of PGN_1808 was detected at a molecular weight higher than 800 kDa (Fig 2C). Additionally, in gel filtration chromatography, the PGN_1808 fraction was eluted at almost the same elution volume as a void volume marker Blue Dextran (molecular weight, 2,000,000) (data not shown), suggesting that PGN_1808 formed a polymer higher than 2,000 kDa. Taken together, these results indicate that PGN_1808 exists as a polymer in bacterial cells, but that it is detergent (SDS) and heat labile compared to FimA and Mfa1 fimbriae.

**Fig 3:**
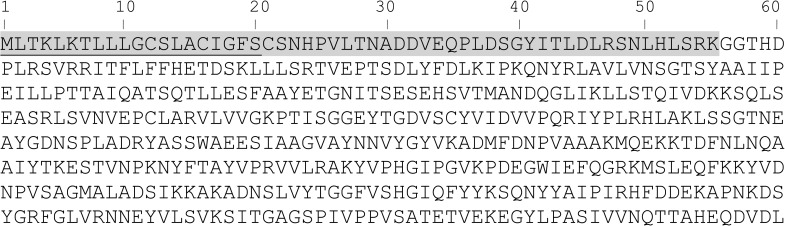
Deduced amino acid sequence of PGN_1808. The LipoP program predicted that the N-terminal 20 amino acids were a signal peptide (underlined). N-terminal sequencing of the 50-kDa PGN_1808 monomer band in the SDS-PAGE analysis (Fig 2A) showed that the N-terminal amino acid was Gly-56, indicating that the N-terminal 55 amino acids were removed for processing (highlighted in gray).

N-terminal sequencing of the PGN_1808 monomer band (shown in Fig 2A) revealed that the N-terminal 55 amino acids were removed (Fig 3). The LipoP program predicted that the 20 N-terminal amino acids of PGN_1808 comprised a signal peptide. FimA and Mfa1 fimbrial components are processed by digestion with trypsin-like proteases, gingipains, which preferentially cleave peptide bonds after Arg or Lys residues and are localized in the outer membrane [26]. We therefore speculate that the newly generated PGN_1808 protein is sorted to the inner membrane by the lipophilic signal peptide, and then to the periplasm, where the signal peptide is removed by a signal peptide peptidase. After further digestion between the 55th (Lys) and 56th (Gly) amino acids likely by gingipains at the outer membrane, the mature form of PGN_1808 polymerizes.

### Fimbriation and cell surface expression of PGN_1808

**Fig 4:**
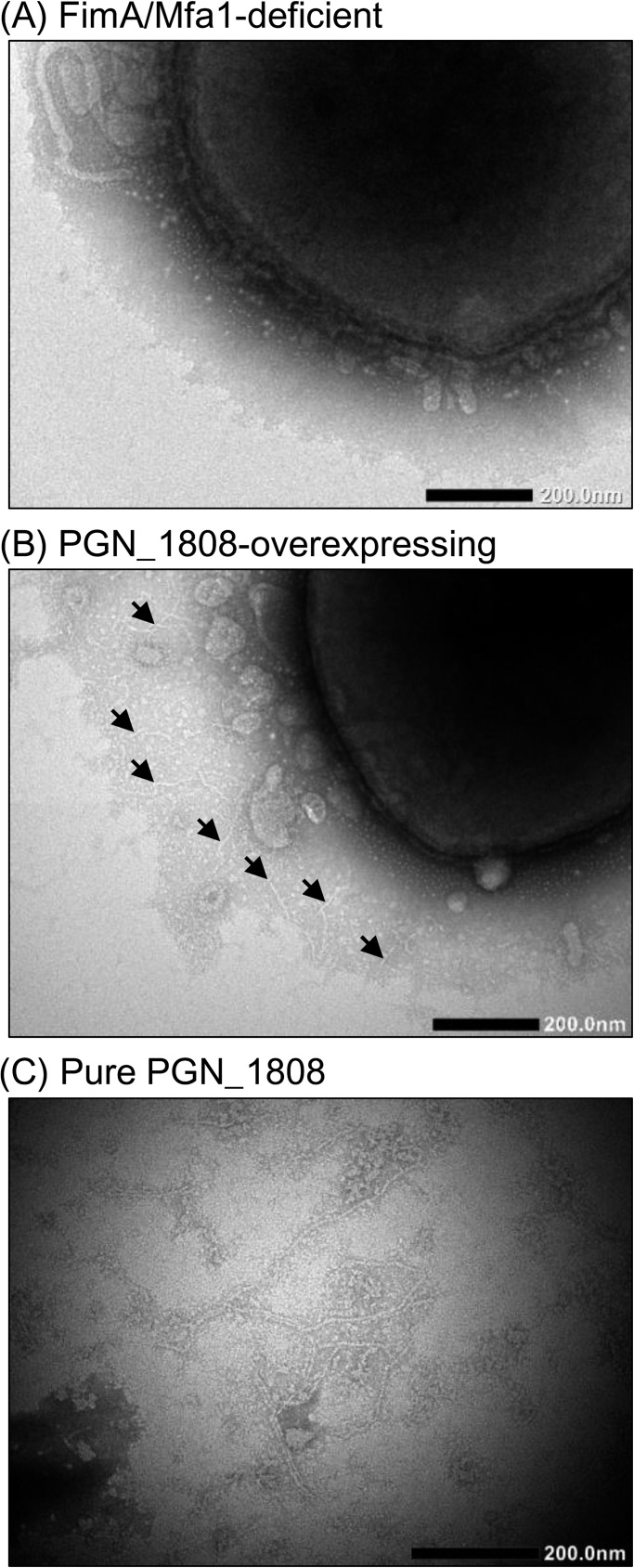
Transmission electron micrographs. Whole cells of the *P*. *gingivalis* mutant that was deficient in both FimA and Mfa1 fimbriae (A), whole cells of the PGN_1808-overexpressing mutant (B), and fractionated PGN_1808 (C) were negatively stained, then observed by TEM. Filamentous structures (2‒3 nm × 200‒400 nm) were observed on cell surfaces of the PGN_1808-overexpressing mutant (indicated by arrows) and in the PGN_1808 fraction. Scale bars indicate 200 nm.

TEM observation did not detect fimbrial structures on bacterial cell surfaces of the *P*. *gingivalis* mutant deficient in both FimA and Mfa1 fimbriae (Fig 4A), as previously reported [18]. Additionally, this strain did not show an agglutination reaction with anti-PGN_1808 serum. On the other hand, the PGN_1808-overexpressing mutant exhibited thin filamentous structures (2‒3 nm × 200‒400 nm) on bacterial cell surfaces (Fig 4B). This strain clearly showed an agglutination with anti-PGN_1808 serum. The filamentous structures were also observed in the PGN_1808 fraction (Fig 4C). These results, taken together with Figs 2 and 4, indicate that PGN_1808 polymerizes to form fimbriae on cell surfaces. However, PGN-1808 appears to be expressed at low levels in wild-type strains, because even the FimA- and Mfa1-deficient mutant did not show detectable fimbrial structures (Fig 4A).

### PGN_1808 expression in *P*. *gingivalis* strains

PGN_1808 was detected in 81 of 84 wild-type strains of *P*. *gingivalis* by western blotting (S2 Table), suggesting that PGN_1808 is generally expressed in *P*. *gingivalis*. We also noticed that the band intensity varied among strains, and that several strains showed a slightly different migration rate in gels (data not shown), indicating that there might be variants of PGN_1808.

### Structure homology-modeling

**Fig 5:**
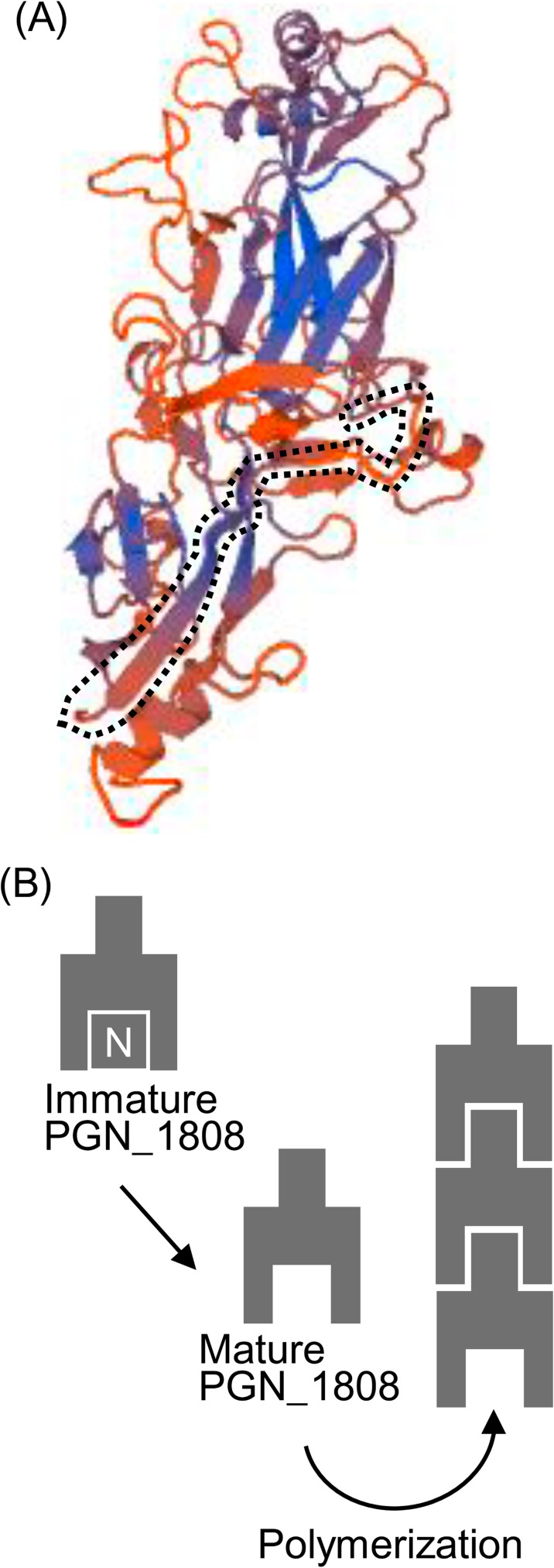
Structure and polymerization models. PGN_1808 without the signal sequence (the N-terminal 20 amino acids) was submitted to structure homology-modeling (A). The color (orange to blue) indicates the QMEAN score, which indicates the reliability of the model (low to high quality, respectively) [23]. The N-terminal sequence (from the 21st to 55th amino acid), surrounded by the black dotted line, was removed for processing (see text). PGN_1808 is predicted to polymerize by donor-strand complementation and cleft-mediated anchorage (B). White letter N denotes the N-terminal region.

We submitted PGN_1808, excluding the predicted signal peptide in the N-terminal 20 amino acids, to structure homology-modeling in the SWISS-MODEL program. The program found that PGN_1808 shared significant homology with a putative cell adhesion protein (BACOVA_01548) from *Bacteroides ovatus* (PDB ID: 4rfj), that was resolved by Xu *et al* [15]. Fig 5A shows the putative structure of PGN_1808, which was constructed based on the BACOVA_01548 structure. The N-terminal sequence from the 21st to 55th amino acid, which was removed for processing as described above, lies in a groove (surrounded by the black dotted line in Fig 5A). The mechanism of fimbrilin assembly is known to involve removal of their N-terminal peptides, creating an extended groove that binds a stretched donor strand of the incoming fimbrilin [27]. The PGN_1808 structure model suggests that the similar mechanism applies to the polymerization of PGN_1808 (Fig 5B).

### Accessory components and transcription unit

We have not yet identified any accessory components of PGN_1808 fimbriae, because no proteins were concomitantly fractionated with PGN_1808. However, this possibility should be addressed in future research using a wild-type strain, rather than the PGN_1808-overexpressing mutant.

Transcription unit comprising *pgn_1808* was examined in *P*. *gingivalis* wild-type strains ATCC 33277 and TDC60 by RT-PCR. We found that *pgn_*1808 was co-transcribed with the upstream genes including *pgn_1805*, *pgn_1806*, and *pgn_1807*, but not with *pgn_1804* and the downstream gene *pgn_1811* (note that *pgn_1809* and *pgn_1810* are absent). The *pgn_1805*, *pgn_1806*, and *pgn_1807* genes are annotated as cysteine-tRNA ligase, patatin (a lipase), and glycosyl transferase, respectively [17]. Further research is needed to elucidate whether there is any functional relationship between PGN_1808 and these molecules.

## Conclusion

We showed that PGN_1808 polymerized to form a fimbria and that it was generally expressed in *P*. *gingivalis*. However, PGN_1808 was detergent and heat labile compared to FimA and Mfa1. The biological function of PGN_1808 fimbriae should be examined in the future.

## Supporting information

S1 Table Primers used for RT-PCR. (DOCX)

S2 Table Fimbrial profile of *P. gingivalis* strains. (DOCX)
