# Supplementary material for: Novel fimbrilin PGN_1808 in Porphyromonas gingivalis
Source: PLoS One. 2017 Mar 15;12(3):e0173541. doi: 10.1371/journal.pone.0173541 (PMC5351860; doi:10.1371/journal.pone.0173541)
Supplement: S1 Table — (DOCX) [file pone.0173541.s001.docx]

S1 Table Primers used for RT-PCR.

| Primer | Sequence (5’ to 3’) | Description |
| --- | --- | --- |
| 1804F | TTGATTACGTTGATTTGGTTATGG | Forward primer annealing to the middle region of *pgn_1804* |
| 1804F2 | CGTTTTCCAGATTCAGCTCATATC | Forward primer annealing to the terminal region of *pgn_1804* |
| 1804R | GCCGACGATATAGGTGGTATTATC | Reverse primer annealing within *pgn_1804* |
| 1805F | TAACAAAGCTTATTCACCCATGAC | Forward primer annealing within *pgn_1805* |
| 1805R | AAAGAGCTGCTATTACCATTGGAC | Reverse primer annealing within *pgn_1805* |
| 1806F | TTTCAAGAATTTCATGGACGATTC | Forward primer annealing within *pgn_1806* |
| 1806R | TACTTGGTACGATCAAACACCTTG | Reverse primer annealing within *pgn_1806* |
| 1807F | TTCTATCCTCGCAGTTTCAATTTG | Forward primer annealing within *pgn_1807* |
| 1807R | AAAGTAACAAAGGAAGCAATGACC | Reverse primer annealing within *pgn_1807* |
| 1808F | AGCAGCGATCTATACAAAAGAATC | Forward primer annealing within *pgn_1808* |
| 1808R | AACTGTATTCCATGACTGACGAAC | Reverse primer annealing within *pgn_1808* |
| 1811F | CCTTCCAATGTAATTTCTCGTTCT | Forward primer annealing within *pgn_1811* |
| 1811R | AAGTATGCCTGAGTCTTTCCTTGT | Reverse primer annealing within *pgn_1811* |

Note that immediately downstream gene of *pgn_1808* is *pgn_1811*. The *pgn_1809* and *pgn_1810* genes are absent.

We confirmed that each gene from *pgn_1804* to *pgn_1811* was transcribed (data not shown).
