# Supplementary material for: Novel fimbrilin PGN_1808 in Porphyromonas gingivalis
Source: PLoS One. 2017 Mar 15;12(3):e0173541. doi: 10.1371/journal.pone.0173541 (PMC5351860; doi:10.1371/journal.pone.0173541)
Supplement: S2 Table — (DOCX) [file pone.0173541.s002.docx]

S2 Table Fimbrial profile of *P. gingivalis* strains^a^.

|  | Strain | Fimbrial type | | |
| --- | --- | --- | --- | --- |
|  |  | FimA^b^ | Mfa1^c^ | PGN_1808^d^ |
| 1 | TDC60 | II | 75 | + |
| 2 | B129 | II | 75 | + |
| 3 | B1 | II | 53 | + |
| 4 | HW24D1 | II | 75 | + |
| 5 | OMZ314 | II | 75 | + |
| 6 | JKG3 | II | 53 | + |
| 7 | 1438 | II | 53 | + |
| 8 | 14018 | II | 75 | + |
| 9 | 14019 | II | 75 | + |
| 10 | 14020 | II | 75 | + |
| 11 | 14021 | II | 75 | + |
| 12 | HU17 | II | 75 | + |
| 13 | Shirai | II | 75 | + |
| 14 | 1439 | II | ND | + |
| 15 | OMZ409 | II | 75 | + |
| 16 | A7A1-28 | II | 53 | + |
| 17 | JKG10 | II | ND | + |
| 18 | B158 | II | ND | + |
| 19 | HG934 | II | 75 | + |
| 20 | TV14 | II | ND |  |
| 21 | 1440 | II | 53 | + |
| 22 | D83T3 | II | 75 | + |
| 23 | Kyudai-3 | II | ND | + |
| 24 | D84B24 | II | 75 | + |
| 25 | JKG6 | II | 75 | + |
| 26 | OUD63 | II | 75 | + |
| 27 | OUD161 | II | 75 | + |
| 28 | Kyudai-4 | II | ND | + |
| 29 | 7680 | II | 75 | + |
| 30 | 6/26 | III | 53 | + |
| 31 | ESO7 | III | 53 | + |
| 32 | ESO9 | III | 53 | + |
| 33 | ESO10 | III | 53 | + |
| 34 | ESO27 | III | 53 |  |
| 35 | ESO51 | III | 53 | + |
| 36 | ESO127 | III | 53 | + |
| 37 | 49417 | III | 53 | + |
| 38 | ESO159 | III | 53 | + |
| 39 | ESO164 | III | 53 | + |
| 40 | ESO187 | III | 53 | + |
| 41 | ESO192 | III | 53 | + |
| 42 | EM3 | III | 75 | + |
| 43 | 19m-1 | Ib | 75 | + |
| 44 | D13B11 | Ib | 75 | + |
| 45 | D67D9 | Ib | 75 | + |
| 46 | 1442 | Ib | 53 |  |
| 47 | En444 | Ib | 53 | + |
| 48 | D96A2 | Ib | 75 | + |
| 49 | JKG9 | Ib | ND | + |
| 50 | ESO132 | Ib | 75 | + |
| 51 | 1436 | Ib | ND | + |
| 52 | D55D13 | Ib | 53 | + |
| 53 | Ando | Ib | 53 | + |
| 54 | MPW1b-01 | Ib | 53 | + |
| 55 | ESO24 | Ib | 75 | + |
| 56 | ESO101 | Ib | 75 | + |
| 57 | ESO75 | Ib | 75 | + |
| 58 | 33277 | I | 75 | + |
| 59 | 27 | I | 75 | + |
| 60 | 381 | I | 75 | + |
| 61 | 1021 | I | 75 | + |
| 62 | 1112 | I | 75 | + |
| 63 | H185 | I | 53 | + |
| 64 | HG405 | I | ND | + |
| 65 | NCTC11834 | I | 75 | + |
| 66 | TDC27 | I | 75 | + |
| 67 | D43B4 | I | 75 | + |
| 68 | JKG4 | Ib | 53 | + |
| 69 | W83 | IV | ND | + |
| 70 | A7436 | IV | 75 | + |
| 71 | SAW3A | IV | 53 | + |
| 72 | B42 | IV | ND | + |
| 73 | En7 | IV | ND | + |
| 74 | Su63 | IV | 53 | + |
| 75 | JKG1 | IV | 75 | + |
| 76 | 222 | IV | ND | + |
| 77 | HG564 | IV | 53 | + |
| 78 | 7692 | IV | 53 | + |
| 79 | HNA99 | V | 53 | + |
| 80 | 244 | V | 53 | + |
| 81 | F1 | V | 53 | + |
| 82 | F2 | V | 53 | + |
| 83 | H184 | V | 53 | + |
| 84 | I2 | V | 53 | + |

^a^ Profiles of FimA and Mfa1 fimbriae had been analyzed in our previous studies (Nagano *et al.*, 2013; Nagano *et al.*, 2015).

^b^ *fimA* genotype.

^c^ 53 and 75 indicate 53-kDa and 75-kDa type of Mfa1 fimbriae, respectively. ND indicate that neither fimbrial protein was detected.

^d^ + showed positive band in western blotting.

**References**

Nagano, K., Abiko, Y., Yoshida, Y., and Yoshimura, F. (2013) Genetic and antigenic analyses of *Porphyromonas gingivalis* FimA fimbriae. *Mol Oral Microbiol* **28**: 392-403.

Nagano, K., Hasegawa, Y., Yoshida, Y., and Yoshimura, F. (2015) A major fimbrilin variant of Mfa1 fimbriae in *Porphyromonas gingivalis*. *J Dent Res* **94**: 1143-8.
